# Virtual screening, molecular dynamics simulations, and *in vitro* analysis of *Sophora flavescens*-derived aloperine against *Haemonchus contortus*

**DOI:** 10.3389/fvets.2025.1620324

**Published:** 2025-06-19

**Authors:** Anben Li, Yan Ma, Wenxi Li, Bintao Zhai, Nana Fu, Jun Li, Qianyu Zhou, Yang Liu

**Affiliations:** ^1^School of Life Sciences, Ningxia University, Yinchuan, China; ^2^Key Lab of Ministry of Education for the Protection and Utilization of Special Biological Resources in Western China, Ningxia University, Yinchuan, China; ^3^Department of Pharmacy, Tongliao People’s Hospital, Tongliao, China; ^4^Key Laboratory of Veterinary Pharmaceutical Development, Lanzhou Institute of Husbandry and Pharmaceutical Sciences, Chinese Academy of Agricultural Sciences, Ministry of Agriculture, Lanzhou, China; ^5^Key Laboratory of Zoonosis Research, Ministry of Education, Institute of Zoonosis, College of Veterinary Medicine, Jilin University, Changchun, China

**Keywords:** *Haemonchus contortus*, virtual screening, aloperine, IVM, HC-Pgp

## Abstract

**Introduction:**

The resistance of *Haemonchus contortus* to ivermectin (IVM) poses a significant economic threat to the global livestock industry. This necessitates alternative strategies for managing the development of drug resistance in *H. contortus*.

**Methods:**

This study employed molecular docking screening, molecular dynamics simulations, and *in vitro* experiments to evaluate the effects of bioactive alkaloids from *Sophora alopecuroides* L. on *H. contortus*.

**Results:**

Molecular docking and dynamics simulations revealed aloperine (ALO)’s strong binding affinity (−6.83 kcal/mol) and stable interaction with HC-Pgp among 13 tested alkaloids. Further evaluation through larval development test (LDT), larval migration inhibition test (LMIT), and scanning electron microscopy revealed that the combined administration of ALO and IVM exerted significantly enhanced inhibitory effects on the development, motility, and morphological integrity of IVM-resistant strains compared to monotherapy groups. Furthermore, the Rhodamine-123 accumulation assay demonstrated that aloperine significantly inhibited HC-Pgp activity (*p* < 0.05).

**Discussion:**

This study provides new perspectives for exploring the natural product ALO as an anthelmintic, HC-Pgp inhibitor, and synergist molecule. Further studies evaluating *in vivo* safety and pharmacokinetic interactions are required to validate these findings.

## 1 Introduction

*Haemonchus contortus*, a pathogenic gastrointestinal nematode that parasitizes the abomasum of ruminants, infects hosts through a complex life cycle. The eggs of *H. contortus* are excreted in host feces and hatch into first-stage larvae (L1), and develop into the second- (L2) and third- (L3) stage larvae within approximately 1 week. The host ingests infective L3 larvae while grazing, after which exsheathed L3 larvae (xL3) progress to fourth-stage larvae (L4), simultaneously acquiring nutrients at the parasitic sites, and maturing into dioecious adults after approximately 3 weeks (1). This can lead to anemia and complications in livestock. In severe cases, it can cause extreme emaciation and even death, representing a significant global disease that results in considerable economic losses (2). Anthelmintics are currently the primary control strategy for these gastrointestinal nematodes, among which ivermectin (IVM) is used as a broad-spectrum, highly effective, low-toxicity, and low-residue macrolide antiparasitic agent. However, the prolonged and improper use of anthelmintics has facilitated the development of drug resistance (3); therefore, the efficacy of anthelmintics warrants further enhancement.

P-glycoproteins (P-gp) are hydrophobic cell membrane proteins with high molecular weight and lipophilicity that belong to the ATP-dependent transport protein family in *H. contortus*. P-gp expel exogenous substances to prevent their accumulation within cells, protecting against toxic molecules (4). P-gp overexpression is one of the mechanisms underlying IVM resistance (5). P-gp are present at various stages of *H. contortus* development, particularly abundant in L3 larvae in structures such as the cuticle (6). A total of 11 functional P-gp genes have been identified in *H. contortus*, namely P-gp 1–4, 9–14, and 16, which are homologous to those in *Caenorhabditis elegans*, except for P-gp16 (7). Inhibition of P-gp expression has been shown to enhance *H. contortus* sensitivity to IVM (8). P-gp inhibitors are classified into four generations based on toxicity scores, with phytochemicals (natural compounds produced by plants), including alkaloids and terpenoids, belonging to the fourth generation (9, 10). Phytochemicals, with their low toxicity and diverse biological properties, show significant potential as P-gp inhibitors and antiparasitic agents (11–13), and are synergistic enhancers for anthelmintics. While they may lack inherent anthelmintic properties, their use alongside anthelmintics enhances the effectiveness of these treatments. However, identifying effective natural HC-Pgp inhibitors is challenging, as extraction and antiparasitic activity evaluation are costly and require specialized equipment. In recent years, the integration of structure-based virtual screening and molecular dynamics simulations has enhanced drug discovery by elucidating the molecular interactions between drugs and their targets. This comprehensive approach helps identify, design, and optimize novel candidate drugs.

*Sophora alopecuroides* L., a plant species in the genus Sophora, belonging to the Fabaceae family, is widely distributed in Western and Central Asia. For example, in China, *S. alopecuroides* L. is primarily found in deserts, dunes, and saline-alkali lands in Ningxia and Xinjiang (14, 15) and has a wide range of medicinal properties, including antiparasitic, antiviral, antibacterial, and neuroprotective (16). Its main chemical components include alkaloids, flavonoids, amino acids, carbohydrates, and organic acids (17). Alkaloids are the primary bioactive components in *S. alopecuroides* L., including aloperine (ALO), matrine, and sophocarpine (18), with various biological properties, such as antioxidant, anticancer, and insecticidal (18, 19). ALO, a quinolizidine alkaloid extracted from *S. alopecuroides* L., is important in mitigating various parasites, such as nematodes and aphids (20–22). Currently, data on ALO in *H. contortus* remain lacking. Therefore, in this study, we determined whether ALO could inhibit P-gps expression and increase the sensitivity of *H. contortus* to IVM. Our findings provide novel insights into the scientific prevention and control of haemonchosis, effective drug use, novel drug development, and promotion of advantageous local resources.

## 2 Materials and methods

### 2.1 Ethics statement

The study design was reviewed and approved by the Animal Ethics Committee of Ningxia University (permit No. 24-F-051). The procedures involving animals were carried out in accordance with the Animal Ethics Procedures and Guidelines of the People’s Republic of China. All efforts were made to minimize suffering and to reduce the number of sheep used in the experiment.

### 2.2 *Haemonchus contortus* strains

The IVM-sensitive and resistant strains of *H. contortus* were provided by the Inner Mongolia Academy of Agricultural and Animal Husbandry Sciences (IMAAAHS). The sensitive strain (HC-S) was isolated from Yuci, Shanxi, China, passaged, and maintained in sheep at IMAAAHS for 6 years. The resistant strain (HC-R) originally came from the Moredun Research Institute in the United Kingdom and was passaged and maintained in sheep at IMAAAHS for 9 years.

### 2.3 Preparation of target proteins

Since the three-dimensional structure of HC-Pgp has not been experimentally resolved, we constructed its structural model using homology modeling. The target protein preparation involved retrieving the full-length amino acid sequence of HC-Pgp (UniProt ID: A0A7I4YQ55) from the UniProt database for homology modeling using SWISS-MODEL.^1^ The constructed protein model underwent rigorous validation through Ramachandran plot evaluation to ensure its suitability for subsequent molecular docking studies. SWISS-MODEL and validation Ramachandran plot ensure structural accuracy of HC-Pgp, enabling reliable virtual screening. Concurrently, Potential ligand binding sites were identified using Schrödinger software (Maestro12.8) suite. Schrödinger’s SiteMap and Glide modules identify ligand-binding pockets, foundational for docking studies. Preprocessing was performed using the Protein Preparation Wizard module. The SiteMap algorithm was applied to detect potential ligand binding sites with a 1.0 Å probe radius, and at least 15 characteristic probes were generated per site. Based on the prediction results, the Glide module was employed to construct a docking grid centered on the primary binding pocket, laying a solid computational foundation for virtual screening.

### 2.4 Preparation of small molecule ligands

The predominant alkaloids in *S. alopecuroides* L. were retrieved from PubChem and subsequently subjected to computational preparation using the LigPrep 5.0 module of Schrödinger Suite. LigPrep adjusts protonation states, removes salts, and generates tautomers/stereoisomers, ensuring ligands mimic biological conditions. To enhance the reliability of docking results, ligand conformations were systematically optimized to mimic interactions under physiological conditions, thereby reducing potential computational false positives. Molecular processing included: (1) adjustment of protonation states under physiological pH conditions (7.0 ± 2.0), (2) removal of inorganic salts and counterions, (3) addition of hydrogen atoms with stereochemical optimization, (4) generation of biologically relevant tautomers and stereoisomers (maximum isomer limit = 32), and (5) conformational optimization through ring-constrained energy minimization employing the OPLS4 force field. OPLS4 force field minimization improves conformational accuracy, enhancing docking reliability. All computational parameters strictly adhered to the module’s default recommended settings to ensure methodological reproducibility and standardization.

### 2.5 Molecular docking screening

Molecular docking were employed to prioritize alkaloids with optimal HC-Pgp binding stability, forming a robust theoretical framework for downstream *in vitro* studies. Initial ADMET filtration of database molecules was performed using the Schrödinger QuickProp module (23), retaining only compounds compliant with Lipinski’s rule of five and devoid of reactive functional groups for subsequent docking studies. Flexible docking was then conducted via the Schrödinger platform to prioritize alkaloids with the highest predicted binding affinities based on docking scores. Interaction analysis of the top five candidate compounds was performed through molecular visualization using Maestro, Discovery Studio (Dassault Systèmes BIOVIA, 2019), and PyMOL to identify key amino acid residues mediating protein-ligand interactions.

### 2.6 Molecular dynamics simulations

Molecular dynamics simulations were conducted using the GROMACS 2022.6 GPU version to confirm dynamic stability and binding mode persistence (24, 25). The initial structure for the simulations was derived from molecular docking results. The ligand topology file was generated employing the AMBER force field through the ACPYPE script, specifically using the AMBER99SB-ILDN force field for the protein topology file. The molecular dynamics simulation protocol included the definition of the simulation box, solvation with water molecules, and the addition of counter ions. Subsequently, energy minimization was performed, followed by equilibration in both the canonical ensemble system (NVT) and the constant-pressure system (NPT). The final phase of the simulation involved 100 ns molecular dynamics run under constant temperature and pressure conditions. Upon completion of the simulation, the last 5 ns of the trajectory were extracted for further analysis. The gmx_mmpbsa tool was utilized for the calculation of free energy to gain insights into the energetics of the molecular system.

### 2.7 Larval development inhibition test

The development inhibition effect was assessed using a modified larval development inhibition test (LDT) (26). In a 24-well plate, 265 μL of physiological saline (Nacl, 0.9%), 70 μL of Earle’s balanced salt solution (Sigma–Aldrich Corporation, United States), 20 μL of *H. contortus* eggs (About 100 eggs), and 5 μL of 5 mg/mLwater-soluble amphotericin B (Solarbio, Beijing, China) were added to each well. The mixture was incubated at 27°C in a biochemical incubator for 24 h. Subsequently, 80 μL of ALO (Baoji Fangsheng Biological Development Co., Ltd., Baoji, China), 80 μL of IVM (Shanghai Yuanye Bio-Technology Co., Ltd., Shanghai, China), a combination of 40 μL ALO + 40 μL IVM, and 1% DMSO (a negative control) was added to the system in individual wells. The final concentrations of both ALO and IVM were prepared through two-fold serial dilutions spanning 0 to 400 ng/mL. Three independent biological replicates were set up for each experimental group. After 6–7 d, L3 larvae were counted under an inverted microscope (OLYMPUS IX73), and the median lethal dose (LD_50_) was calculated using the Karber method, its basic arithmetic formula is:lgLD_50_ = ∑1/2 (Xi + Xi + 1) (Pi + 1 − Pi). Where Xi is the logarithm of the dose Pi is the rate of developmental inhibition.

### 2.8 Larval migration inhibition test

Feces from lambs infected with *H. contortus* were collected and incubated in aerobic conditions at 24°C with 80–85% humidity for 5–10 d. L3 larvae were isolated from the feces using the Baermann funnel system (27), and the migration ability was assessed using a modified larval migration inhibition test (LMIT) (28). In each well, approximately 100 L3 larvae were added to 1,710 μL of sterile water. Various treatment solutions were then added to individual larval suspensions: 90 μL of ALO (final concentration 0.39–400 μg/mL), 90 μL of IVM (final concentration 0.39–400 μg/mL), 45 μL of IVM + 45 μL of ALO, and 1% DMSO (a negative control). Three replicate experiments were conducted for each treatment.

After 24 h of incubation at 24°C, the liquid from each well was transferred to migration plates containing 0.125% agar and incubated for an additional 24 h. The solutions at the bottom of the migration plates were transferred to a new 24-well plate. The number of migrating larvae was assessed under an inverted microscope, and the migration rate was calculated. Using GraphPad Prism 8.0.1, a regression curve was fitted with the logarithm of each treatment concentration and the larval migration inhibition rate on the X-and Y-axes, respectively. The half-maximal effective concentration (EC_50_) was calculated using the equation: Y = Bottom + (Top − Bottom)/(1 + 10^[(LogEC50 – X) × HillSlope]).

### 2.9 Evaluation of morphological changes

The exsheathed L3 larvae were exposed to 25 μg/mL ALO, 25 μg/mL IVM, or a combination of 25 μg/mL ALO + 25 μg/mL IVM for 24 h, with 0.1% DMSO serving as the vehicle control. Post-treatment, the specimens were fixed with 2.5% glutaraldehyde and morphologically analyzed using a scanning electron microscope (SU8100) at an accelerating voltage of 3.0 kV.

### 2.10 Rhodamine 123 determinations

The effect of ALO on HC-Pgp activity was determined by monitoring rhodamine 123 (Rhe123) accumulation in *H. contortus* (29). Approximately 30,000 exsheathed L3 larvae were exposed to ALO, IVM, or their combination for 24 h (as described previously), followed by incubation in Rhe123 solution (1 mL of 1.5 μM) under dark conditions at room temperature using a constant temperature shaker for 30 min. The worms were subsequently centrifuged (3,000 × g, 3 min) and washed twice with 1 mL distilled water. The pellet was resuspended in 1 mL distilled water and maintained in darkness for 60 min. After final centrifugation (3,000 × g, 3 min), the supernatant was collected and stored protected from light for 60 min prior to analysis. Rhe123 concentration in the supernatant was quantified using a multifunctional microplate reader (Agilent Technologies Inc., United States) with specific fluorescence parameters (excitation wavelength λex = 495 nm, emission wavelength λem = 525 nm). Sample concentrations were calculated against a standard curve. Three independent experimental replicates were performed. Statistical analysis was conducted using GraphPad Prism 8.0.1, with statistical significance defined as *p* < 0.05.

## 3 Results

### 3.1 Homology modeling of HC-Pgp

Using the primary amino acid sequence of HC-Pgp (accession number: A0A7I4YQ55) as the target, a BLASTP and SWISS-MODEL template search identified 4f4c.1. A (X-ray, 3.40 Å) as the best template with a sequence identity of 55.83% with HC-Pgp, which is sufficient to construct a reliable model. The sequence alignment between HC-Pgp and 4f4c.1. A is illustrated in Supplementary Figure S1. The model generated using SWISS-MODEL was validated through a Ramachandran plot (Supplementary Figure S2). The analysis revealed that 93.3% of the residues were in the most favored regions and 6.6% in the additional allowed regions, while 0.1% of the amino acids were in the disallowed regions. These data validated the modeled structure, making it suitable for docking studies (30).

### 3.2 Screening of HC-Pgp inhibitors from major alkaloids in *Sophora alopecuroides* L.

**Figure 1:**
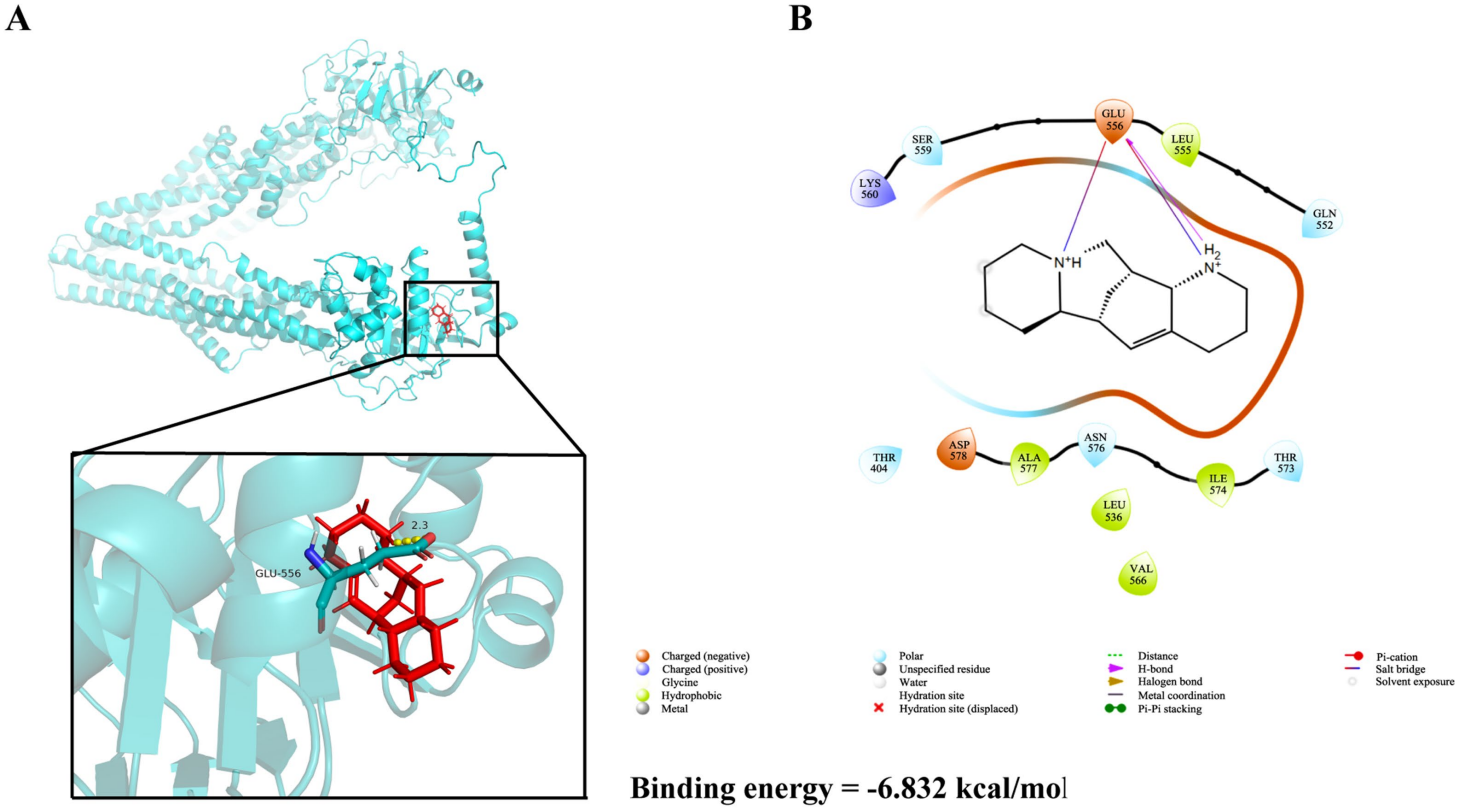
The three-dimensional and two-dimensional ligand interaction patterns between ALO and amino acid residues of the HC-Pgp receptor. **(A)** Three-dimensional molecular interaction between ALO and HC-Pgp. **(B)** Two-dimensional molecular interaction between ALO and HC-Pgp.ALO, aloperine.

Structure-based virtual screening (SBVS) in drug discovery offers multiple advantages, including the capability to efficiently screen large compound libraries, reduced costs and time compared to experimental screening, as well as the potential to explore broad chemical spaces. In this study, preliminary ADMET filtering of database molecules was performed using the QuickProp module, retaining molecules compliant with Lipinski’s rule of five (Ro5) and devoid of reactive fragments. The results demonstrate that all 13 major alkaloids from *S. alopecuroides* L. exhibited physicochemical parameters within acceptable ranges for hydrogen bond donors/acceptors, molecular weight, and lipophilicity (Table 1), suggesting their potential as promising drug candidates. Subsequent molecular docking screening revealed that ALO exhibited the highest binding affinity (−6.83 kcal/mol) toward HC-Pgp (Supplementary Table S1; Supplementary Figure S3). As shown in Figures 1A,B, ALO interacts with HC-Pgp through binding interactions predominantly mediated by hydrogen bonding and hydrophobic forces at the Glu556 residue.

**Table 1:** Prediction of ADME properties for 13 *Sophora alopecuroides* L. alkaloids.

Compounds	MW	H-bond donors	H-bond acceptors	QplogPo/w	QPlogHERG
Aloperine	232.37	1.00	3.50	1.88	−4.69
Anagyrine	244.34	0.00	5.00	1.46	−4.42
Cytisine	190.24	1.00	4.50	0.67	−4.01
Isomatrine	248.37	0.00	5.00	0.82	−2.21
Lupanine	248.37	0.00	5.00	0.92	−2.29
Matrine	248.37	0.00	5.00	0.85	−2.22
N-Methylcytisine	204.27	0.00	5.00	0.79	−4.25
Oxymatrine	264.37	0.00	6.00	0.93	−1.64
Oxysophocarpine	262.35	0.00	6.00	1.48	−3.25
Sophocarpine	246.35	0.00	5.00	1.45	−3.97
Sophoramine	244.34	0.00	5.00	1.39	−4.16
Sophoridine	248.37	0.00	5.00	0.84	−2.41
Sparteine	234.38	0.00	4.00	1.87	−4.48

MW: Molecular weight; QPlogPo/w: Predicted octanol/water partition coefficient; QPlogHERG: Predicted IC50 value for blockage of the HERG K + channels.

### 3.3 Construction between ALO and HC-Pgp is highly stable

**Figure 2:**
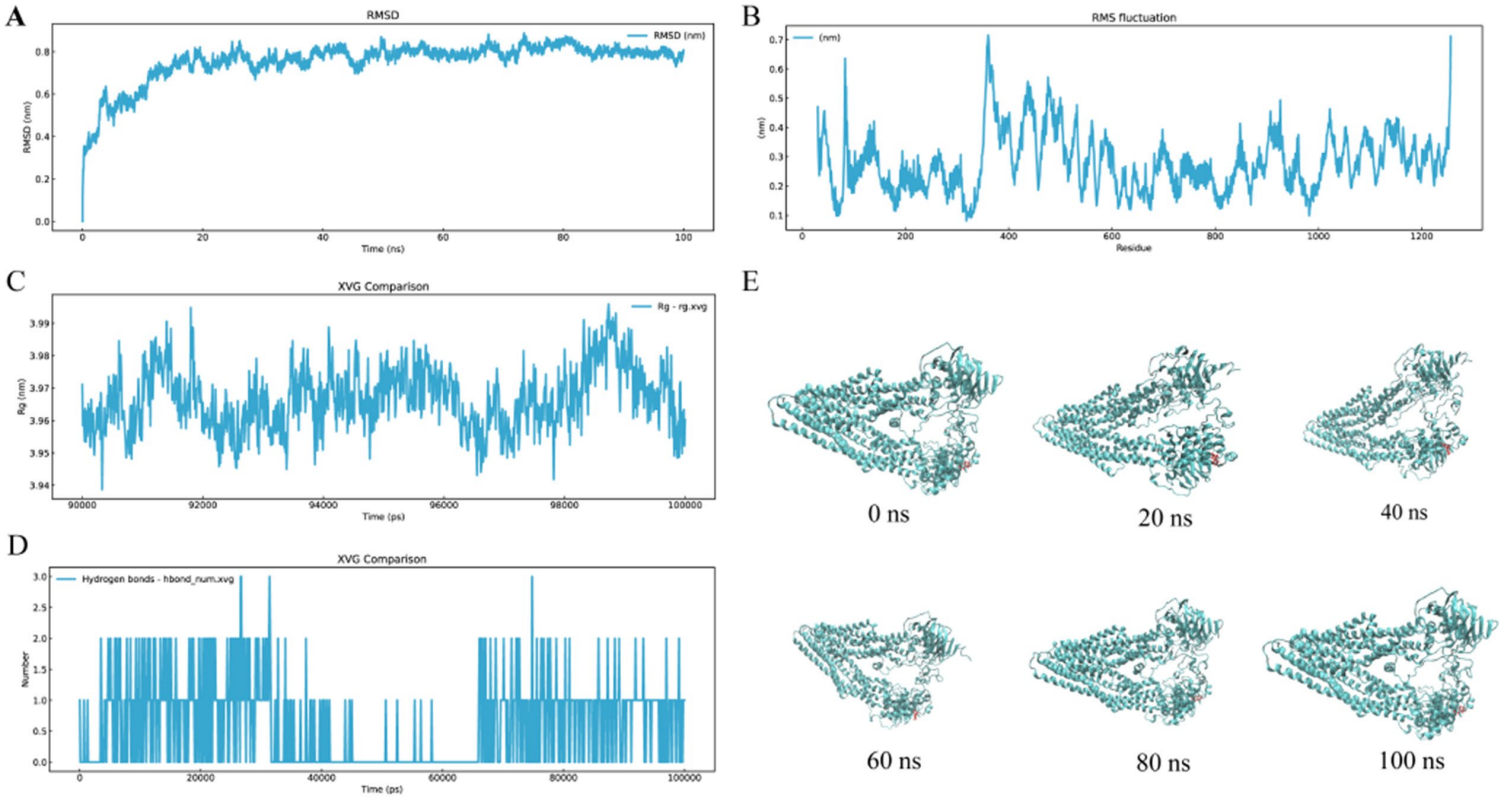
Molecular dynamic interactions of ALO with HC-Pgp receptors in 100 ns. **(A)** RMSD. **(B)** RMSF. **(C)** Rg curves. **(D)** H-bond number of the peptide-receptor complexes. **(E)** Binding mode evolution of the ALO-HC-Pgp complex at 0, 20, 40, 60, 80, and 100 ns. ALO, aloperine.

Molecular dynamics simulations spanning 100 ns were conducted to evaluate the interaction between ALO and HC-Pgp. As shown in Figure 2A, the ALO-HC-Pgp complex achieved equilibrium after 20 ns of simulation. RMSF analysis, which quantifies atomic deviations from average positions, revealed relatively low values (<1 Å) across both ligand and receptor domains (Figure 2B), indicating sustained structural stability. The Rg, calculated as the mass-weighted root mean square distance of atoms from the system’s centroid, demonstrated minimal variation (ΔRg ≈ 0.05 nm) within the range of 3.94–3.99 nm throughout the trajectory (Figure 2C), further confirming conformational stability. Protein-ligand hydrogen bonding patterns, critical for binding affinity and complex stabilization, were dynamically monitored (Figure 2D). Additionally, Figure 2E depicts the binding mode of the ALO-HC-Pgp complex at 0, 20, 40, 60, 80, and 100 ns, illustrating the initial movement of the ALO around the binding pocket of the HC-Pgp. These multi-parameter analyses demonstrate that ligands achieve specific regulation of HC-Pgp function by stabilizing active site conformations and forming high-strength hydrogen bond networks.

### 3.4 Binding free energy analysis

**Figure 3:**
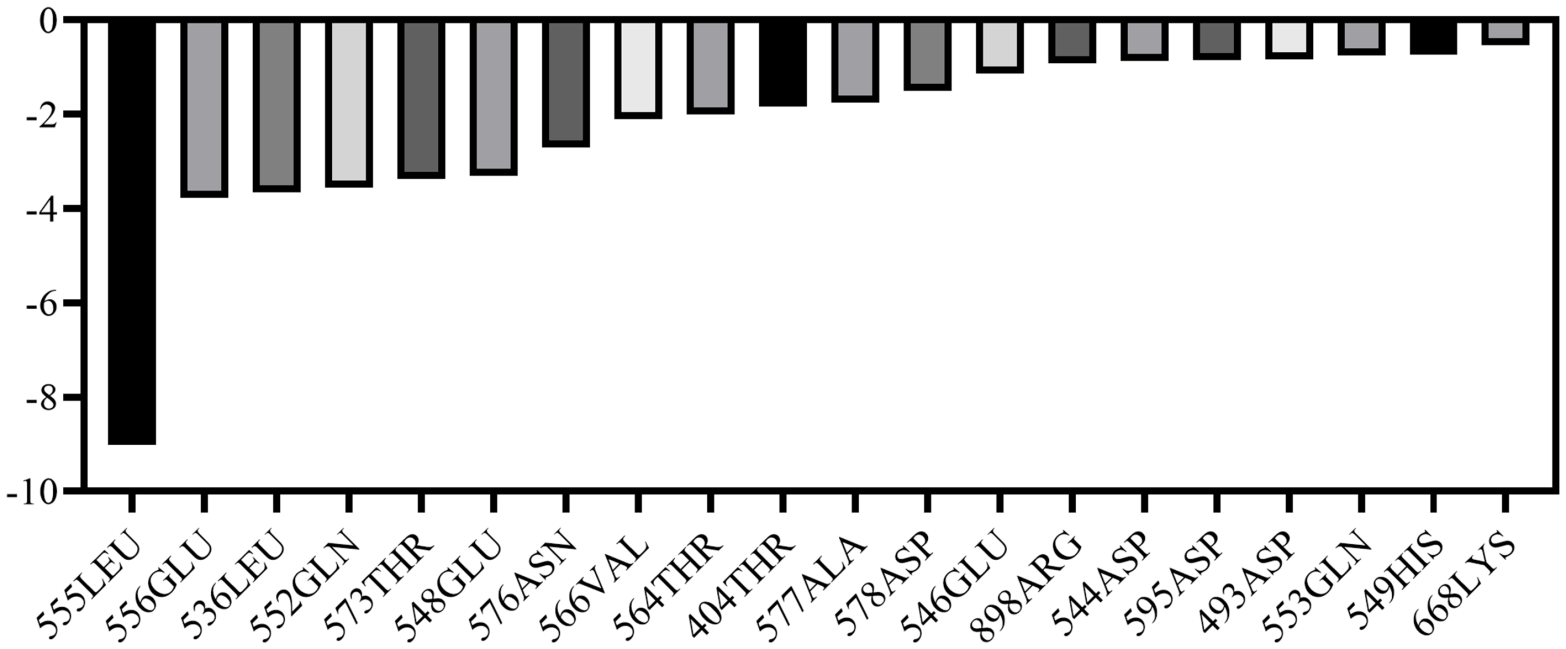
Energy decomposition of key amino acid residues in HC-Pgp during ligand binding (top 10 contributors).

MM-PBSA calculations were performed using the final 5 ns of molecular dynamics simulations to quantify the binding affinities between ligand and its receptor. Van der Waals energy, electrostatic energy, electrostatic contribution to solvation-free energy from the Poisson-Boltzmann model, nonpolar contribution, and entropy were calculated for each complex. The results demonstrated that ALO exhibits strong binding affinity with HC-Pgp, as evidenced by a calculated binding free energy of −88.16 kcal/mol (Table 2). Furthermore, we analyzed the residue binding energy contribution of each amino acid in the receptor after ligand binding, which is a critical approach for identifying hotspot residues that play major roles in ligand binding during simulations. The energy decomposition results revealed that LEU555 and GLU556 in the active site and catalytic residues made significant contributions, while residues such as LEU536, GLN552, THR573, and GLU548 also played important roles in energy contributions (Figure 3).

**Table 2:** MM-PBSA based average binding free energy of HC-Pgp with ALO in kj/mol.

Interaction force	Binding free energy (kJ/mol)
Van der waals	−146.04
Electrostatic	−37.71
Polar solvation	89.68
Nonpolar solvation	−18.08
ΔH	−112.14
-TΔS	23.98
Total binding energy	−88.16

### 3.5 Effects of ALO on *Haemonchus contortus* development

**Figure 4:**
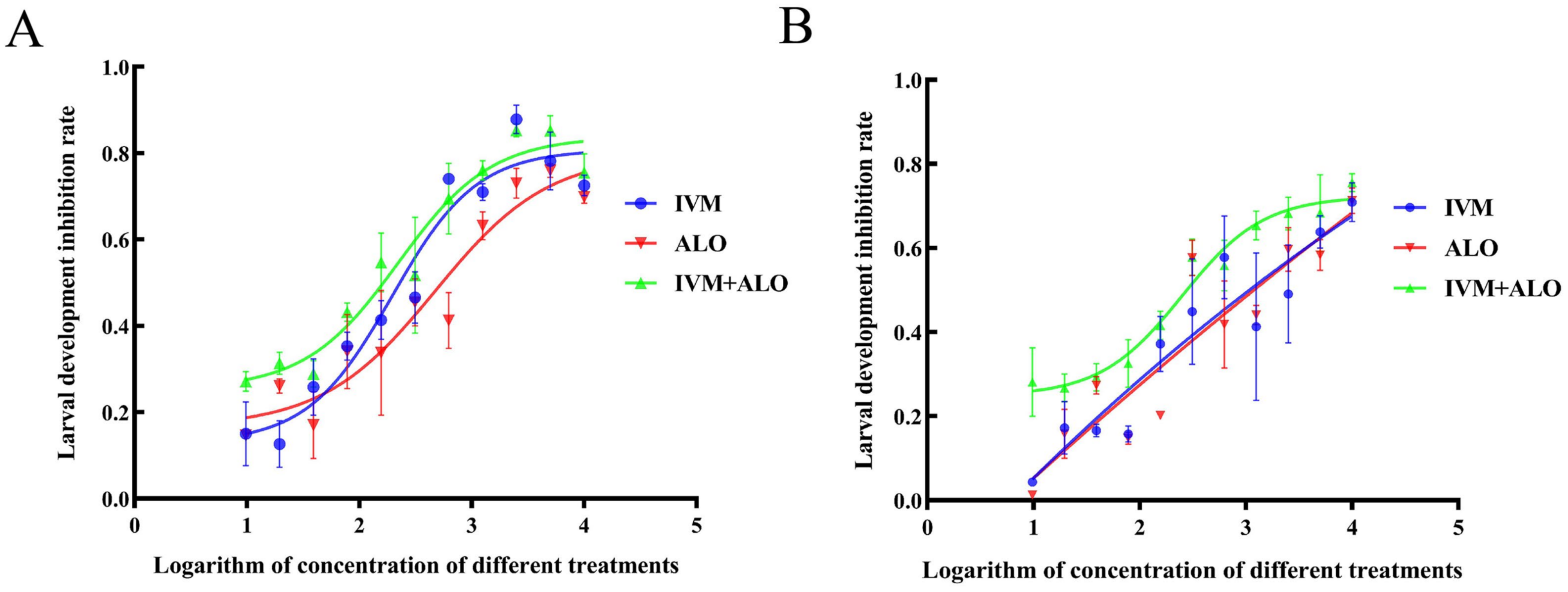
Inhibitory effect of ALO alone or in combination with IVM on the larval development of sensitive strain **(A)** and resistant strain **(B)** of *Haemonchus contortus*. ALO, aloperine.

A comparison of the median lethal dose between the experimental and control groups in the LDT revealed an inhibitory effect of ALO on larval development to a lesser extent than that of IVM. The ALO and IVM combination exerted a significant inhibitory effect on *H. contortus* development that was approximately 2-to 3-fold of the effect of ALO treatment alone (Table 3; Figure 4). In the HC-R strain, the LD_50_ for the ALO + IVM group was 17.72 ng/mL, while the LD_50_ values for the IVM and ALO groups were 45.02 and 50.90 ng/mL, respectively, indicating that the ALO + IVM combination had a significantly higher developmental inhibition effect on HC-R than that of either agent alone.

**Table 3:** LD_50_ results of ALO alone or in combination with IVM on *Haemonchus contortus*.

Treatment	LD_50_ (ng/mL) and 95% CI	
	HC-S	HC-R
IVM	15.25 [8.73, 26.65]	45.02 [28.55, 70.99]
ALO	19.13 [12.53, 29.21]	50.90 [30.04, 83.47]
IVM + ALO	9.97 [5.24, 18.95]	17.72 [11.14, 28.20]

ALO: aloperine; IVM: ivermectin; LD_50_: median lethal dose; HC-S: sensitive strain; HC-R: resistant strain; 95% CI: 95% confidence interval.

Similarly, in the HC-S strain, ALO exhibited an inhibitory effect on *H. contortus* development; ALO and IVM combination showed a significantly higher inhibitory effect on HC-S development than ALO or IVM monotherapy.

### 3.6 Effects of ALO on the migration ability of *Haemonchus contortus*

**Figure 5:**
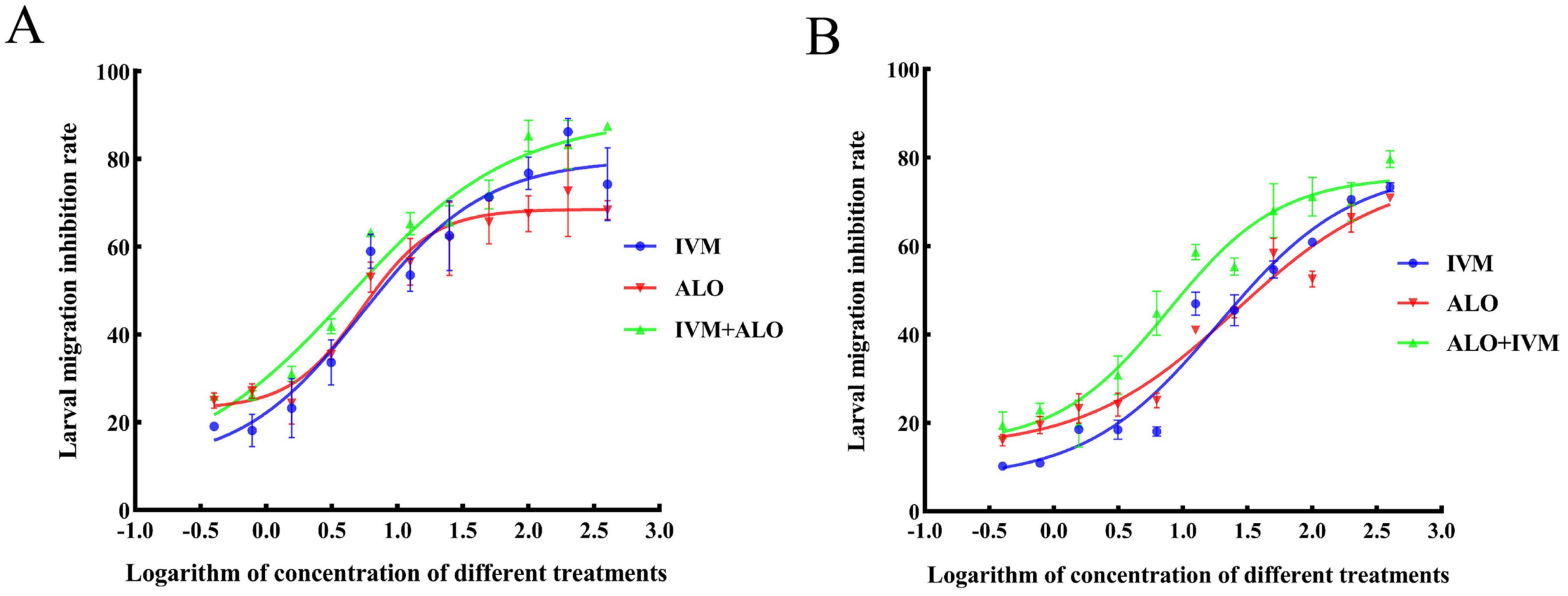
Inhibitory effect of ALO alone or in combination with IVM on the migration of the sensitive **(A)** and resistant **(B)** strains of *Haemonchus contortus*. ALO, aloperine.

At the L3 stage of larvae development, the LMIT method was used to evaluate the effect of the ALO and IVM combination on the migration ability of *H. contortus* (Table 4; Figure 5). The EC_50_ values for the resistant strain of *H. contortus* were 25.43 μg/mL for ALO, 18.77 μg/mL for IVM, and 7.50 μg/mL for the combination of ALO and IVM. For the sensitive strain, the EC_50_ values were 5.33 μg/mL for ALO, 5.42 μg/mL for IVM, and 4.34 μg/mL for the combination of ALO and IVM. These results indicate that ALO can inhibit the migration ability of both IVM-resistant and sensitive strains of *H. contortus*. Furthermore, the ALO and IVM combination showed a significantly greater inhibitory effect on the migration ability of the HC-R strain than ALO or IVM alone, although this effect was not significant for the HC-S strain. This differential effect appears to be directly associated with P-gp, as evidenced by the Rhe123 accumulation assay, which revealed significantly higher activity in HC-R compared to HC-S (*p* < 0.05) (Supplementary Figure S4).

**Table 4:** EC_50_ results of ALO alone or in combination with IVM on *Haemonchus contortus*.

Treatment	EC_50_ (μg/mL) and 95% CI	
	HC-S	HC-R
IVM	5.42 [0.89, 10.50]	18.77 [12.24, 38.66]
ALO	5.33 [3.62, 7.80]	25.43 [15.00, 293.20]
IVM + ALO	4.34 [0.11, 8.03]	7.50 [4.66, 11.37]

ALO: aloperine; IVM: ivermectin; EC_50_: effective concentration for 50% inhibition; HC-S: sensitive strain; HC-R: resistant strain; 95% CI: 95% confidence interval.

### 3.7 Effects of ALO on the morphology of *Haemonchus contortus*

**Figure 6:**
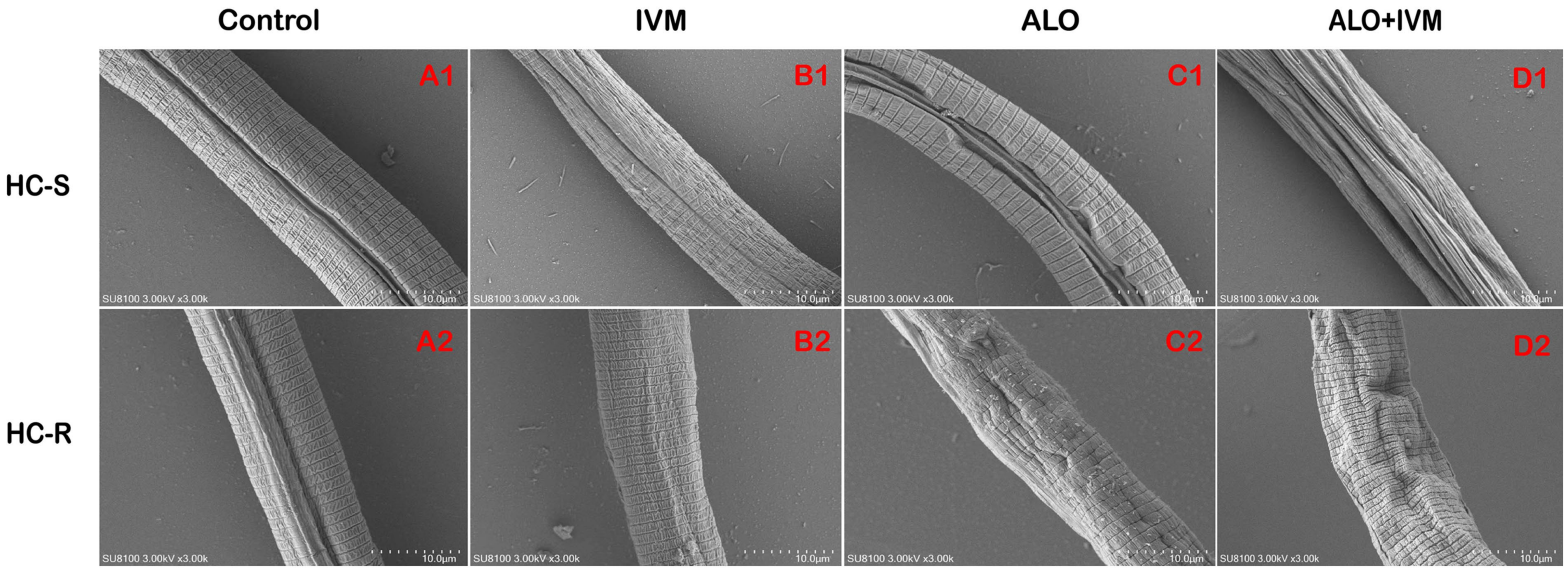
Effect of ALO alone or in combination with IVM on the morphology of *Haemonchus contortus*. **(A1)** HC-S control group, **(B1)** HC-S IVM group, **(C1)** HC-S ALO group, **(D1)** HC-S ALO + IVM group, **(A2)** HC-R control group, **(B2)** HC-R IVM group, **(C2)** HC-R ALO group, and **(D2)** HC-R ALO + IVM group. ALO, aloperine.

The nematode cuticle, a complex extracellular matrix composed of proteins, lipids, and carbohydrates, protects nematodes from environmental and pathogenic damage (31). To evaluate the effects of ALO on the cuticle of *H. contortus* L3, we observed *H. contortus* L3 exposed to 25 μg/mL ALO under a scanning electron microscope. ALO-induced changes in the cuticle and led to severe cuticle shrinkage when combined with IVM (Figure 6). This is consistent with LDT and LMIT results, indicating that the ALO and IVM combination has a greater effect on the cuticle of *H. contortus* than either agent individually, suggesting that ALO has a synergistic effect with IVM.

### 3.8 ALO modulate *Haemonchus contortus* Pgps activity

**Figure 7:**
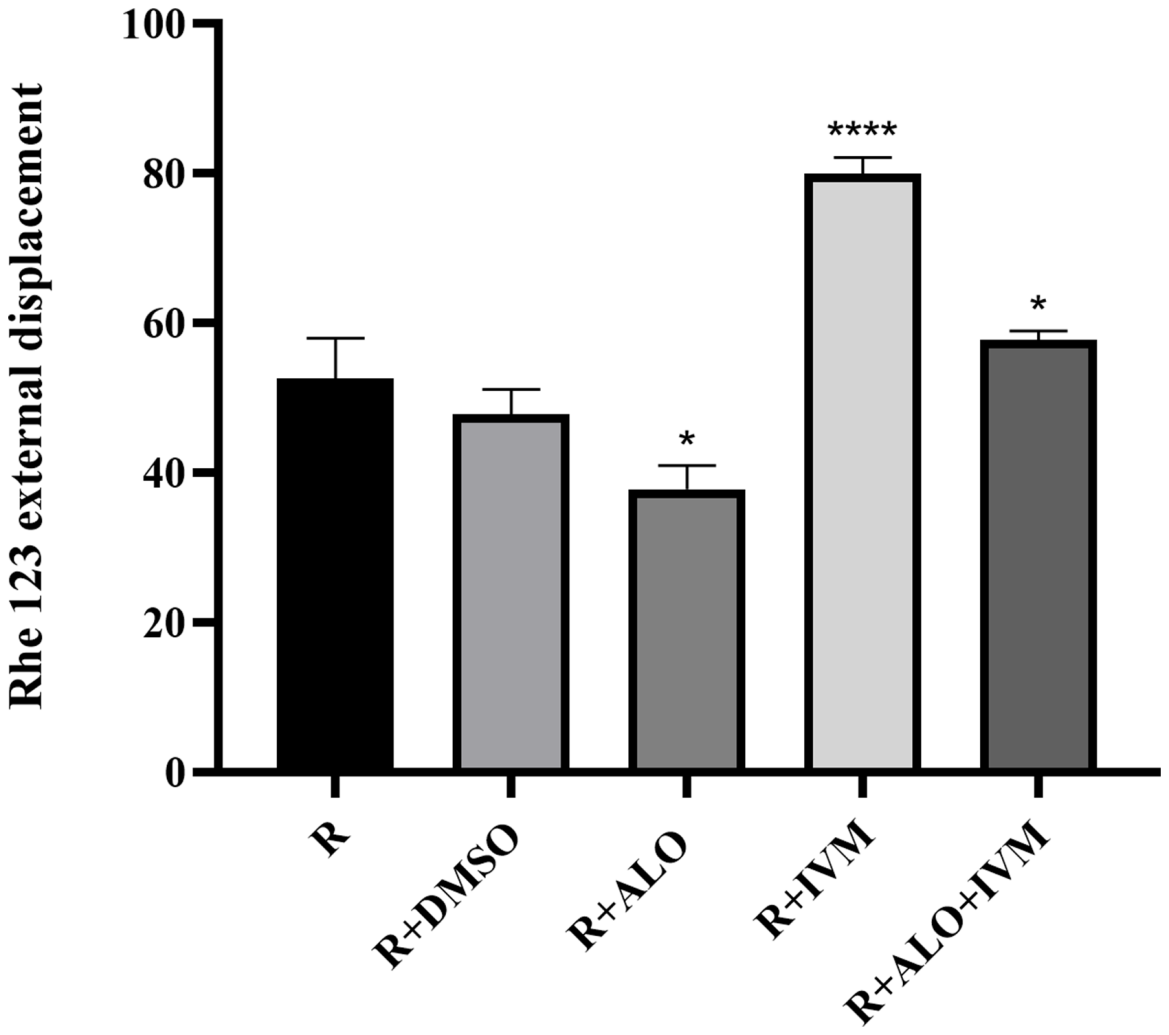
Effect of ALO on rhodamine 123 accumulation. Rhodamine 123 (R, 1.5 μM) was treated with Rhodamine 123 (R, 1.5 μM) alone or in combination with DMSO (1%), picloram, IVM, or AIO + IVM for 24 h. Data are expressed as mean ± SEM (*n* = 3). Statistical significance compared to the R-alone group was determined by one-way ANOVA with Tukey’s *post hoc* test (*p < 0.05, ****p < 0.0001).

The intracellular accumulation of Rhe 123 among different treatment groups was analyzed to evaluate the effects of ALO on HC-Pgp activity (Figure 7). Compared with the control group (R + 1%DMSO), the R + 25 μg/mL IVM group exhibited a significantly higher intracellular Rhe 123 concentration (*p* < 0.05), indicating reduced efflux activity or enhanced retention. In contrast, the R + 5 μg/mL ALO group displayed a significantly lower Rhe 123 concentration relative to the control (*p* < 0.05). Notably, the combination treatment group (R + 5 μg/mL ALO + 25 μg/mL IVM) showed a pronounced synergistic interaction, with Rhe 123 concentrations being significantly lower than those in the R + IVM group alone (*p* < 0.05). These results suggest that ALO effectively modulates HC-Pgp-mediated efflux activity, potentially reducing substrate efflux.

## 4 Discussion

Members of the ABC transporter superfamily, particularly P-gp, have been implicated in this phase III detoxification pathway through their capacity to actively export MLs from parasitic cells (5, 32). P-gps are active in all life stages of *H. contortus* (7), and their expression inhibition increases the sensitivity of *H. contortus* to IVM (8, 33–35). Plant extracts, as fourth-generation P-gp inhibitors, enhance the efficacy of existing anthelmintics, such as IVM (36). Data on whether ALO, an alkaloid extracted from *S. alopecuroides* L., can inhibit the expression of P-gp and enhance the efficacy of IVM are lacking.

Computer models serve as essential resources, with ligand-based methods, such as quantitative structure–activity relationships, and structure-based methods, such as molecular docking, widely used for predicting biological activities and screening potential natural product drugs (37, 38). These approaches offer a rapid and economical solution for identifying P-gp inhibitors or substrates (39, 40). A computer-aided drug–molecule docking model has been developed by docking anthelmintics with the Cel-Pgp-1 molecule, with confirmed applicability (36, 41). Here, we selected P-gp as a potential target for molecular screening. We hypothesized that alkaloids in *S. alopecuroides* L. bind to P-gps, thereby enhancing the efficacy of IVM against *H. contortus*. Molecular dynamics simulations revealed that ALO could bind to HC-Pgp with great stability via van der Waals forces and hydrogen bonds. Identifying the docking site will facilitate further structural modifications of the natural product ALO to develop novel, effective P-gps inhibitors.

The inhibitory function of alkaloids on P-gp is attributed to a basic nitrogen atom and two planar aromatic rings. Piperidine and quinoline alkaloids inhibit P-gp expression (42, 43). ALO is a natural compound containing two fused piperidine rings with trivalent nitrogen atoms and is a derivative of piperidine alkaloids (44). In the binding conformation of ALO with HC-Pgp, the basic nitrogen atoms and planar aromatic rings of ALO interact with Glu 556 through hydrogen bonds and van der Waals forces (Figure 1), thereby altering the spatial conformation of HC-Pgp, blocking its interaction with substrates, and affecting its biological activities. Our study observed that ALO could significantly reduce HC-Pgp expression in the resistant strain of *H. contortus* and was not significantly different from the expression of P-gp in the sensitive strain. Therefore, ALO is a potential HC-Pgp modulator that enhances the sensitivity of *H. contortus* to IVM.

*In vitro* tests are used in preliminary evaluations of the anthelmintic effects of plant extracts (45). In these studies, eggs or larvae of *H. contortus* are directly exposed to plant extracts to assess their effects on eggs and larval development and motility. This study observed that ALO could aid in controlling gastrointestinal nematodes in livestock, with anthelmintic activities affecting the motility, development, and cuticle of *H. contortus*. Meanwhile, the ALO and IVM combination could enhance the effect of IVM against *H. contortus*. However, previous studies have reported that ALO and lupinine, which share structural similarity, could exhibit significant insecticidal activity by binding to receptors for nicotinic acetylcholine and acetylcholine (21, 46). These collective findings suggest that ALO exerts its anthelmintic and synergistic effects via multimodal mechanisms involving multiple molecular targets, rather than being solely dependent on P-gp interactions. Future studies will investigate the specific molecular targets of ALO in *H. contortus* and validate the contribution of P-gp-mediated pathways to the observed synergism, thereby elucidating the mechanistic basis of ALO-IVM combinatorial efficacy. Meanwhile, *in vivo* experiments and adult-stage studies are crucial for validating drug efficacy. Therefore, in subsequent work, animal model experiments and pharmacodynamic evaluations at the adult stage will be conducted, with a focus on pharmacokinetics and *in vivo* safety.

This study is the first to demonstrate the inhibitory effects of ALO extracted from a region-specific medicinal plant against *H. contortus* and its synergistic effects with IVM, indicating its potential for enhancing the efficacy of IVM. The findings of this study offer important insights for the prevention and control of haemonchosis, appropriate drug usage, new drug discovery, and the development and use of plant resources.

## Data Availability

The original contributions presented in the study are included in the article/Supplementary material, further inquiries can be directed to the corresponding authors.

## Footnotes

^1^ https://swissmodel.expasy.org/
