# Supplementary material for: Virtual screening, molecular dynamics simulations, and in vitro analysis of Sophora flavescens-derived aloperine against Haemonchus contortus
Source: Front Vet Sci. 2025 Jun 19;12:1620324. doi: 10.3389/fvets.2025.1620324 (PMC12221915; doi:10.3389/fvets.2025.1620324)
Supplement: Supplementary file 1 [file Table_1.docx]

***Supplementary Material***

**1 Supplementary Figures and Tables**

**1.1 Supplementary Figures**

**
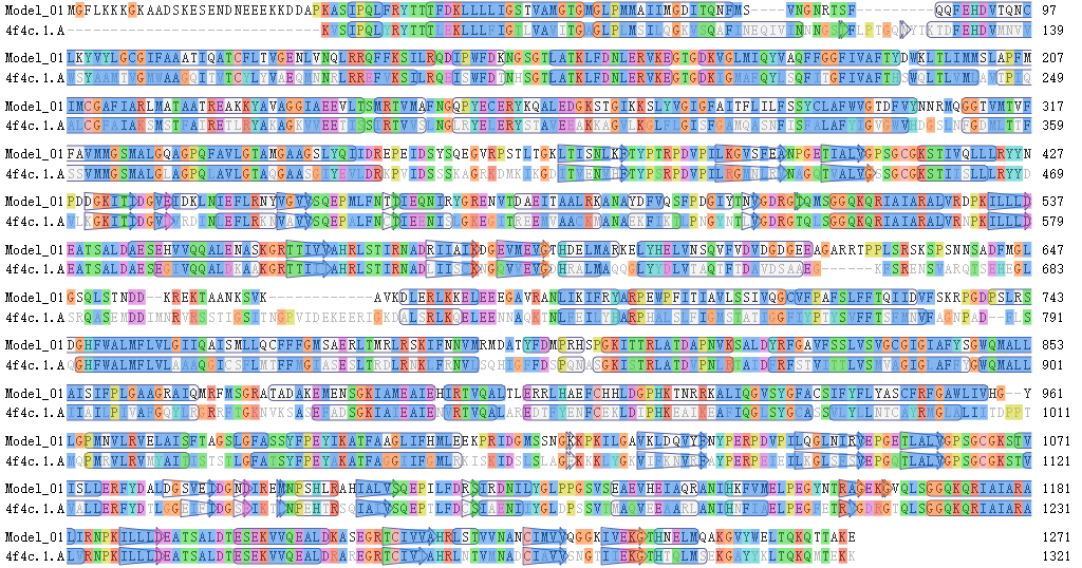
**

**Supplementary Figure 1.** Sequence comparison of HC-Pgp with 4f4c.1.A. model.01 for HC-Pgp


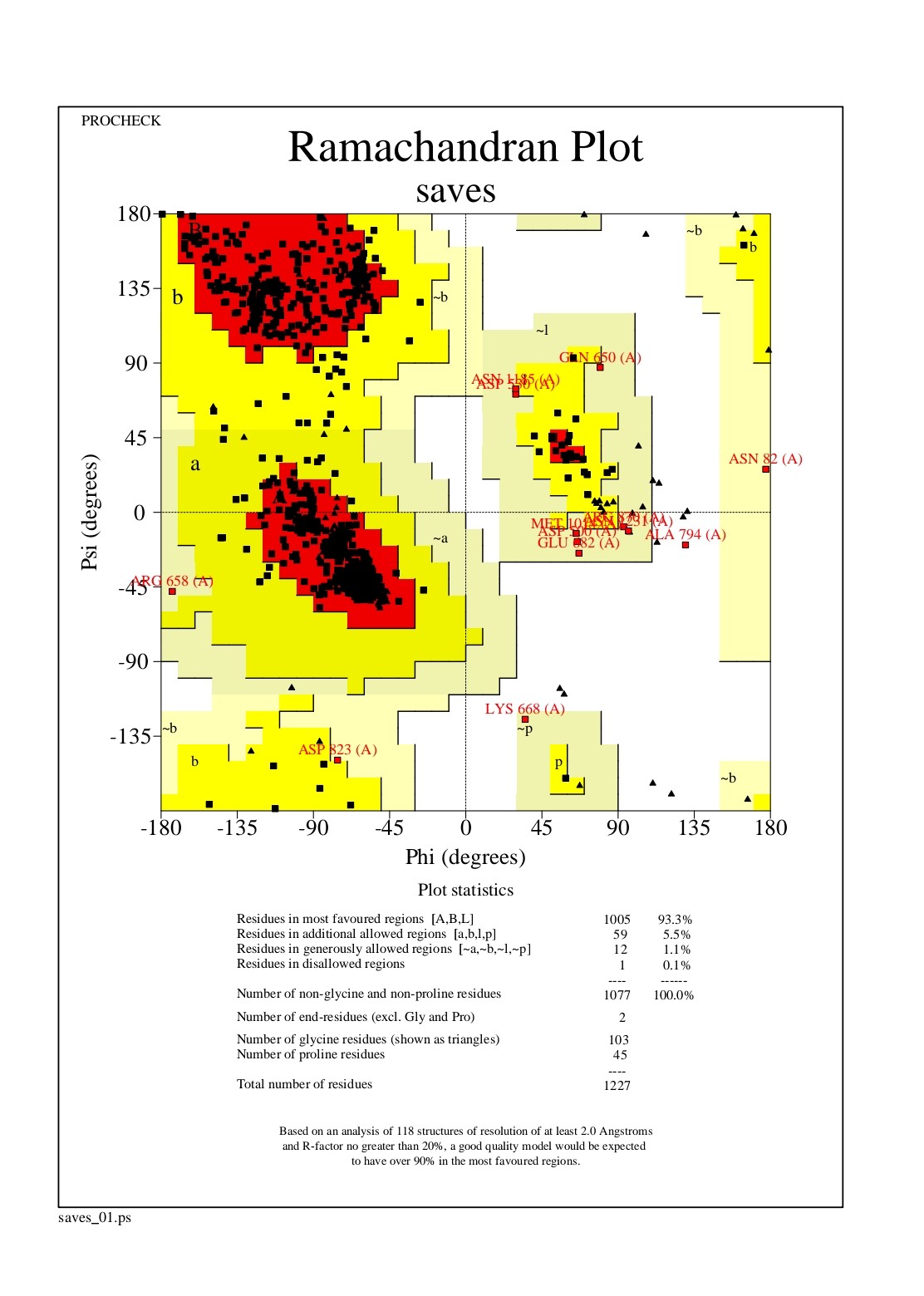


**Supplementary Figure 2.** Validation of modeled structure of HC-Pgp protease using Ramachandran plot.


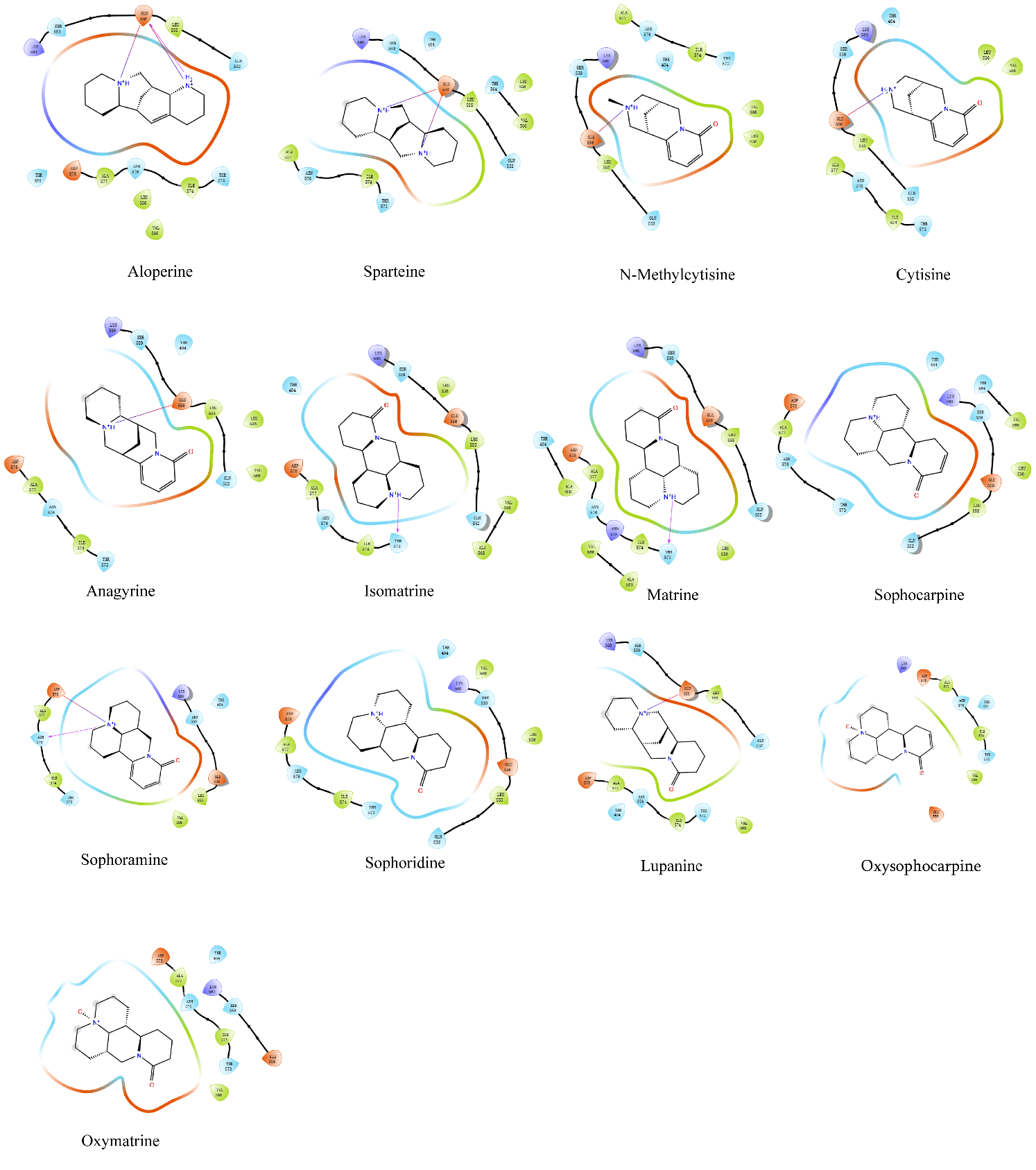


**Supplementary Figure 3.** 2D molecular interactions of ALO with HC-Pgp protein.


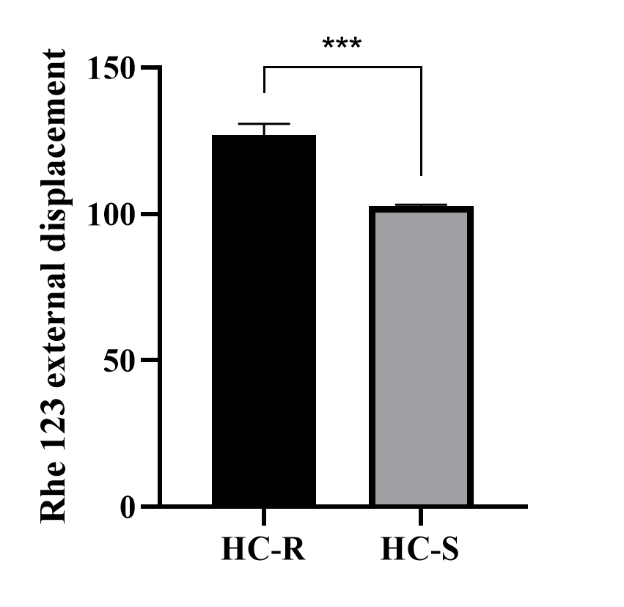


**Supplementary Figure 4.** P-gp activity of HC-R vs HC-S. Data are expressed as mean ± SEM (n = 3). Statistical significance compared to the R-alone group was determined by T teset .(**p* < 0.05, ***p* < 0.01).

**1.2 Supplementary Table**

**Supplementary table 1.** Molecular Docking Results of 13 Sophoridine Compounds with HC-Pgp.

| Compounds | docking score |
| --- | --- |
| [Aloperine](https://pubchem.ncbi.nlm.nih.gov/compound/162147) | -6.832 |
| Sparteine | -6.617 |
| N-Methylcytisine | -6.299 |
| Cytisine | -6.134 |
| Anagyrine | -6.121 |
| Isomatrine | -5.969 |
| Verapamil | -5.755 |
| Matrine | -5.635 |
| Sophocarpine | -5.5 |
| Sophoramine | -5.422 |
| Sophoridine | -5.389 |
| Lupanine | -5.36 |
| Oxysophocarpine | -3.407 |
| Oxymatrine | -3.239 |
